# Supplementary material for: Stochastic fluctuations of bosonic dark matter
Source: Nat Commun. 2021 Dec 16;12:7321. doi: 10.1038/s41467-021-27632-7 (PMC8677790; doi:10.1038/s41467-021-27632-7)
Supplement: Supplementary file 1 — Supplementary Information [file 41467_2021_27632_MOESM1_ESM.pdf]

Supplementary material

Supplementary Note 1. CALCULATING THE EXCLUSION PLOT REVISION FACTOR

Ultralight dark matter fields are composed of a large number of individual modes with randomized phases. Thus, as noted in the main text, due to the central-limit theorem, such virialized ultra light fields (VULFs) belong to a broad class of Gaussian random fields that are encountered in many subfields of physics. The statistical properties of such fields are fully described by the two-point correlation functions in the time domain or power-spectral densities in frequency space. A VULF-specific discussion is presented in Ref. [1].

Here we consider ultralight dark matter searches in the regime where the observation time T is shorter than the coherence time τ_c of dark matter field. Because the VULF oscillations are nearly coherent on these short time scales, the VULF-generated signal $s(t)$ in a detector can be described by an oscillatory function

$$s(t) = \gamma\xi\phi(t) = \gamma\,\xi\,\Phi_0\,\cos(2\pi f_\phi t + \theta)\,. \tag{1}$$

Here f_ϕ is the field frequency (specific to a particular realization of the VULF as the Compton frequency, f_c, is Doppler shifted by the relative motion with respect to the detector, $f_\phi = f_c + m_\phi v^2/(2h)$), θ is an unknown yet fixed phase, ξ is a constant determined by the details of the experiment, and γ is a coupling strength that is, assuming a null result, to be constrained by the measurement. We fix the amplitude of the DM field to be positive, $\Phi_0 \geq 0$ and the phase θ of the field to lie in the range $[0 - 2\pi)$. The coupling strength γ can have an arbitrary sign.

In the conventional approach, the DM field amplitude Φ_0 is fixed to its root-mean-square value Φ_{DM}, defined in the main main body of the paper. We refer to such an approach as *deterministic*. The key issue is that for a given experimental run $T \ll \tau_c$, the value of the VULF amplitude Φ_0 can substantially differ from Φ_{DM}; the VULF amplitude randomly fluctuates. This is apparent from the simulation presented in Fig. 1 of the main text. Thus Φ_0 has to be treated as a nuisance parameter in the analysis. We refer to this treatment as *stochastic*. This has important implications for establishing constraints on γ.

The probability densities for the DM field amplitude in the two approaches can be summarized as

$$p(\Phi_0|\Phi_{\mathrm{DM}}) = \begin{cases} \delta(\Phi_0 - \Phi_{\mathrm{DM}})\,, & \text{deterministic} \\ \frac{2\Phi_0}{\Phi_{\mathrm{DM}}^2}\exp\left(-\frac{\Phi_0^2}{\Phi_{\mathrm{DM}}^2}\right)\,, & \text{stochastic} \end{cases}\,. \tag{2}$$

The stochastic probability distribution for the field amplitude can be recast into the probability distribution for the local energy density $\rho = \Phi_0^2 f_c^2/2$ (where f_c is the Compton frequency, not the Doppler shifted f_ϕ) of the dark matter field,

$$p(\rho|\rho_{\mathrm{DM}})d\rho = \frac{1}{\rho_{\mathrm{DM}}}e^{-\rho/\rho_{\mathrm{DM}}}d\rho\,, \tag{3}$$

in agreement with the distribution used in Ref. [2].

As an analytically treatable case, we assume that the data stream was acquired on a uniform time grid. Considering the oscillating nature of the signal (1), we will work in frequency space, applying a discrete Fourier transform (DFT) to the data. The data set is assumed to include N measurements, taken over the total observation time T. To make the derivation as transparent as possible, we assume that the intrinsic instrument noise is white with variance σ^2. The assumption of white noise, however, is hardly necessary, and the derivation remains valid for the more general case of colored noise. The generalization can be accomplished by replacing $N\sigma^2$ with the noise power spectral density $\tilde{S}(f_p)$, where f_p is the DFT frequency. We use DFT notation and definitions of Ref. [1] (see appendix of that paper for a review of Bayesian statistics in frequency space).

Suppose we would like to establish constraints on the coupling strength γ at one of the DFT frequencies $f_p = f_\phi$ with $\tilde{d}_p$ being the DFT component of the original data. A commonly used test statistic is the excess-power statistic [3] and we will use its Fourier amplitude analog, $A_p = |\tilde{d}_p|/\sqrt{N\sigma^2}$, see Sec. Supplementary Note 3 for a more detailed discussion of the likelihood. Note that θ becomes important when sampling time T is short when compared to $1/f_c$, which we treat in Sec. Supplementary Note 1 C. The likelihood at f_p (we now drop the unnecessary p subscript) is then

$$\mathcal{L}\left(A|A_s\right) = 2Ae^{-A^2-A_s^2}I_0\left(2AA_s\right)\,, \tag{4}$$

for a given excess-signal amplitude $A_s = |\tilde{s}_p|/\sqrt{N\sigma^2}$ with $\tilde{s}_p = N\gamma\xi\Phi_0 e^{i\theta}/2$ being the DFT component of the signal (1) at $f_p = f_\phi$ and I_0 being the modified Bessel function of the first kind. This likelihood is general and in agreement

with those used in other broadband searches [1, 4]. In a more general case this likelihood entails a product over the relevant frequency space bins, and over multiple channels of a network [1, 4, 5].

The likelihood is the starting point of most, if not all, inference frameworks and here we will demonstrate several approaches using Eq. (4). We approach the problem from a Bayesian perspective and derive the posterior distribution for γ in the stochastic and deterministic cases using both a uniform and Jeffreys' prior in Sec. Supplementary Note 1 A. We discuss the subtle difficulties in the use of the uniform prior for this particular problem and the benefits of utilizing objective priors. In Sec. Supplementary Note 1 B we demonstrate a marginal likelihood approach to compare the two cases, where the results are also briefly discussed for the case of the gradient coupling in Sec. Supplementary Note 1 C.

As discussed in Refs. [6–10], the exclusion limit can depend on the chosen analysis method. We discuss several approaches in the subsequent sections. Crucially in our case, Lindley's paradox (disagreement between Bayesian and frequentist limits), is resolved with the use of the Berger-Bernardo reference prior. It is important to emphasize that, regardless of the chosen analysis, accounting for the stochastic behavior generically relaxes constraints on couplings of VULFs to standard model particles and fields.

A. Bayesian approach

Bayesian inference of some parameter of interest, y, requires prior information, $p(y)$, to derive the respective posterior distribution, $p(y\big|D)$, given the data D. If there is a nuisance parameter z, marginalization of the joint posterior is performed to remove it using

$$p(y\big|D) = \int_z p(y,z\big|D)dz. \tag{5}$$

Given the likelihood $\mathcal{L}(D\big|y,z)$, application of Bayes theorem yields the joint posterior

$$p(y,z\big|D) = \frac{p(y,z)\mathcal{L}(D\big|y,z)}{p(D)} = \frac{p(y,z)\mathcal{L}(D\big|y,z)}{\int_z\int_y p(y,z)\mathcal{L}(D\big|y,z)dzdy}, \tag{6}$$

where the denominator is a normalization constant. With the joint prior $p(y,z) = p(y)p(z\big|y)$ and Eq. (5), it is equivalent to applying Bayes' theorem directly to the marginal likelihood

$$p(y\big|D) \propto p(y)\mathcal{L}_m(D\big|y), \tag{7}$$

with the marginal likelihood defined as

$$\mathcal{L}_m(D\big|y) = \int_z \mathcal{L}(D,z\big|y)dz = \int_z \mathcal{L}(D\big|y,z)p(z\big|y)dz. \tag{8}$$

For our particular problem, the parameter of interest is the coupling strength γ, the nuisance parameter is Φ_0, and the observed data are A. We will compare the resulting limits on γ using the two distributions for Φ_0 shown in Eq. (2). The shape of the resulting posterior distribution $p(\gamma\big|A)$ has immediate implications for placing constraints, also in the event of a DM detection, as the stochastic nature of the VULF affects uncertainty in the value of γ. Here, we assume that the dark matter signal is well below the detection threshold, an assumption consistent with current experimental results. Then the constraint at the 95% credible interval (the Bayesian analog to the confidence interval), $\gamma_{95\%}$, can be determined from

$$\int_0^{\gamma_{95\%}} p(\gamma\big|A)d\gamma = 0.95\,. \tag{9}$$

The correction factor obtained will be a ratio of the constraints determined from the deterministic and stochastic Φ_0 distributions, $\gamma_{95\%}^{\rm stoch}/\gamma_{95\%}^{\rm det}$. For the remainder of this section we derive constraints in terms of the excess signal amplitude A_s where the results can be converted into those of γ. We note that working in terms of Fourier amplitude implies our constraint is actually on $\big|\gamma\big|$, if we worked in the complex Fourier domain the coupling could be negative and a factor of two would be introduced to Eq. (9).

Applying Bayes theorem, Eq. (6), to our likelihood, Eq. (4) the posterior distribution for the excess signal amplitude, A_s is

$$p(A_s\big|A) \propto p(A_s)2Ae^{-A^2-A_s^2}I_0\left(2AA_s\right), \tag{10}$$

where the exact shape of the posterior, and resulting limits, depend strongly on the choice of prior $p(A_s)$. The requirement of this prior is the main departure from frequentist or purely likelihood based approaches. While a uniform prior can seem a natural and intuitive choice for an unknown model parameter (all values are equally likely), it can lead to a number of issues that can be avoided with the use of objective priors [11, 12]. Objective Bayesian statistics derive prior distributions using formal rules instead of basing them off of degree of belief. Importantly, these objective priors can ensure invariance of the inference results under a change of variables (e.g. between power and amplitude), see Ref. [13] for a review. We demonstrate this lack of invariance and a resulting improper posterior when using a uniform prior after a change of variables to power in Sec. Supplementary Note 1 A 2.

1. *Berger-Bernardo method*

In this section we demonstrate the Berger-Bernardo reference prior for the case of a separation of the parameters as discussed above, with a parameter of interest and nuisance parameter y and z respectively [13, 14]. First, we find the marginal likelihood as defined in Eq. (8) for our particular case using the stochastic $p(\Phi_0|\Phi_{\rm DM})$

$$\mathcal{L}_m\left(A\middle|A_s^{\rm DM}\right) = \int_{\Phi_0} \mathcal{L}\left(A\middle|A_s^{\rm DM}, \Phi_0\right) p(\Phi_0|\Phi_{\rm DM}) d\Phi_0 = 2Ae^{-A^2/(1+A_s^{\rm DM\,2})}/(1+A_s^{\rm DM\,2}), \tag{11}$$

with $A_s^{\rm DM} = A_s|_{\Phi_0=\Phi_{\rm DM}} = \sqrt{N}|\gamma\xi|\Phi_{\rm DM}/(2\sigma)$, the excess signal power evaluated at $\Phi_0 = \Phi_{\rm DM}$. Note that $\mathcal{L}\left(A\middle|A_s^{\rm DM}, \Phi_0\right)$ is Eq. (4) with $A_s \to A_s^{\rm DM}\Phi_0/\Phi_{\rm DM}$.

This marginal likelihood is then used to calculate the Berger-Bernardo reference prior, which happens to be equivalent (now that there are no more nuisance parameters [14]) to Jeffreys' prior

$$p(A_s^{\rm DM}) \propto \sqrt{I(A_s^{\rm DM})}, \tag{12}$$

where $I(A_s^{\rm DM})$ is the Fisher information,

$$I(A_s^{\rm DM}) = -\int \mathcal{L}_m(A|A_s^{\rm DM}) \frac{\partial^2}{\partial A_s^{\rm DM\,2}} \log \mathcal{L}_m(A|A_s^{\rm DM}) dA, \tag{13}$$

giving the prior (please note that this is effectively a prior on γ given the definition of $A_s^{\rm DM}$)

$$p(A_s^{\rm DM}) \propto A_s^{\rm DM}/(1+A_s^{\rm DM\,2}). \tag{14}$$

We can now use this prior to solve for our posterior distribution

$$p_{\rm stoch}(A_s^{\rm DM}|A) = p(A_s^{\rm DM})\mathcal{L}_m(A|A_s^{\rm DM})/p(A) = \frac{2A^2A_s^{\rm DM}e^{-A^2/\left(1+A_s^{\rm DM\,2}\right)}}{\left(1+A_s^{\rm DM\,2}\right)^2\left(1-e^{-A^2}\right)}. \tag{15}$$

The corresponding 95% credible interval, Eq. (9), is found assuming A is at the experimental detection threshold, see Sec. Supplementary Note 1 B, $A^{\rm dt} = \sqrt{\ln(1/\alpha)}$ where $\alpha = 0.05$ is the type-I error (a false negative: i.e., rejecting the hypothesis when it is actually true)

$$\gamma_{95\%}^{\rm stoch} = 15.6\frac{\sigma}{\sqrt{N}\xi\Phi_{\rm DM}}. \tag{16}$$

For the deterministic case we use the other distribution of Eq. (2) which is equivalent to setting $A_s \to A_s^{\rm DM}$ in the original likelihood, Eq. (4). Then the deterministic posterior distribution is found using the same Berger-Bernardo reference prior (as we note below, this is technically incorrect but has little effect on the results)

$$p_{\rm det}(A_s^{\rm DM}|A) \propto p(A_s^{\rm DM})\mathcal{L}(A|A_s^{\rm DM}) \propto \frac{A_s^{\rm DM}e^{-A_s^{\rm DM\,2}}I_0(2AA_s^{\rm DM})}{1+A_s^{\rm DM\,2}}. \tag{17}$$

Please see Fig. 2 in the main text for a comparison of Eq. (15) and Eq. (17).

Numerical integration under the same assumptions as before, $A = A^{\rm dt}$, yields a constraint at the 95% credible interval of

$$\gamma_{95\%}^{\rm det} = 5.28\frac{\sigma}{\sqrt{N}\xi\Phi_{\rm DM}}. \tag{18}$$

Comparing the deterministic result with Eq. (16) derived in the stochastic treatment, we find

$$\gamma_{95\%}^{\rm stoch} \approx 3.0\,\gamma_{95\%}^{\rm det}\,. \tag{19}$$

We note that this factor is sensitive to the choice of A and would be reduced if $A = \langle A \rangle$ rather than $A^{\rm dt}$ was chosen.

Rigorously, one should calculate the Berger-Bernardo reference prior for the deterministic likelihood (which would be Jeffreys' prior, Eq. (12), using Eq. (4) with $A_s \to A_s^{\rm DM}$). However, this prior does not have an analytic form and the deterministic constraint is relatively insensitive to the choice of prior. Using the aforementioned prior, the stochastic Berger-Bernardo prior, or a uniform prior all yield constraints that agree within 6.3% of Eq. (18). For completeness, we note that using a uniform prior, numerical integration can be avoided resulting in the deterministic posterior

$$p_{\rm det}(A_s^{\rm DM}|A) = \mathcal{L}(A|A_s^{\rm DM})/p(A) = \frac{2e^{-(A^2/2+A_s^{\rm DM\,2})}I_0(2AA_s^{\rm DM})}{\sqrt{\pi}I_0(A^2/2)}. \tag{20}$$

This shows that the choice between the three priors has minimal effect on the constraint when using the deterministic model for field amplitude. However, as will be clear in the next section, this insensitivity to the chosen prior is not true for the stochastic case.

2. *Stochastic case for uniform and objective priors*

While the deterministic case is insensitive to the choice of prior, we will show that this is not true for the stochastic case. Repeating the calculation done in Eq. (15) to calculate the stochastic posterior but with a uniform prior,

$$p_{\rm stoch,uniform}(A_s^{\rm DM}|A) \propto \mathcal{L}_m(A|A_s^{\rm DM}), \tag{21}$$

yields a constraint with numerical integration of

$$\gamma_{95\%}^{\rm stoch,uniform} = 63.2\frac{\sigma}{\sqrt{N}\xi\Phi_{\rm DM}}, \tag{22}$$

and a correction factor of ~ 13 which is considerably different from the result using the Berger-Bernardo method, Eq. (19).

A serious problem with the uniform prior is revealed by analyzing the effect of a change of variables on the resulting constraint. In order for the results to be meaningful and consistent, the constraint should be independent of the choice to work in amplitude or power (as is the case in the frequentist approach discussed in the next section). In particular, using the uniform prior after a change of variables to excess power, $P = |\tilde{d}_p|^2/(N\sigma^2)$,

$$p_{\rm stoch,uniform}(P_s^{\rm DM}|P) = \frac{\mathcal{L}_m(P|P_s^{\rm DM})}{\int \mathcal{L}_m(P|P_s^{\rm DM})dP_s^{\rm DM}}, \quad \text{with} \quad \mathcal{L}_m(P|P_s^{\rm DM}) = e^{-P/(1+P_s^{\rm DM})}/(1+P_s^{\rm DM}) \tag{23}$$

reveals a denominator that does not converge, yielding an improper posterior distribution. See Eq. (45) in Sec. Supplementary Note 3 for the likelihood in terms of excess power P. Use of an objective prior addresses exactly this issue. Using the Berger-Bernardo method shown above, Eq. (12), in the context of the excess-power test statistic gives the prior

$$p(P_s^{\rm DM}) = 1/(1 + P_s^{\rm DM}), \tag{24}$$

where we again note that this is effectively a prior on γ with $P_s^{\rm DM} = P_s|_{\Phi_0=\Phi_{\rm DM}} = N\gamma^2\xi^2\Phi_{\rm DM}^2/(4\sigma^2)$.

Using this prior yields the constraint

$$\gamma_{95\%}^{\rm stoch} = 15.6\frac{\sigma}{\sqrt{N}\xi\Phi_{\rm DM}}, \tag{25}$$

agreeing within a fraction of a percent with the result obtained for the analysis using amplitude. For this reason, we find that the effect of the stochastic nature of the VULF on experimental constraints is most reasonably estimated in the Bayesian framework by using the objective prior. Furthermore, as we show in the next section, the effect of stochastic fluctuations of the VULF on constraints analyzed using a frequentist approach yields results similar to that obtained in the Bayesian framework employing the objective prior in Sec. Supplementary Note 1 A 1.

B. Frequentist approach using marginal likelihood

A frequentist constraint can be obtained via hypothesis testing and using the Type-I error (or false alarm rate), α, and Type-II error (or false detection rate), $1-\beta$ (see Ref. [15] or more recent editions). Type-I error is the rejection rate of the null hypothesis when it is true while the Type-II error is the acceptance (or non-rejection) of the null hypothesis when an alternative (signal) hypothesis is true. We illustrate a critical (exclusion) region and threshold that matches these two errors in Fig. 1.

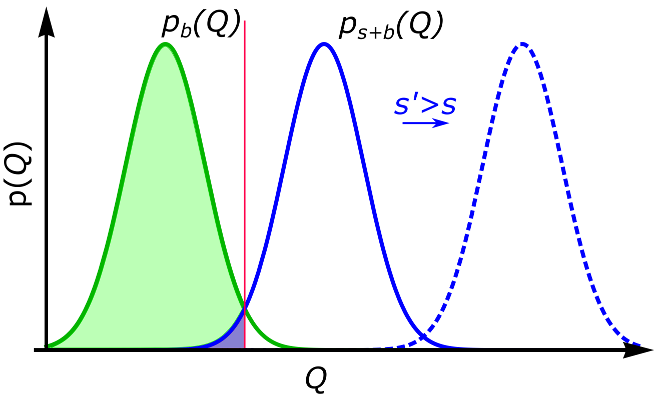

Supplementary Figure 1: Illustrative figure of a hypothesis test for background vs. signal plus background. The figure represents PDFs of the test statistic Q for various values of the signal parameter. The green curve represents the background only hypothesis and the blue curves signal values $s>0$. The Type-I error, α, and detection threshold, red line, are set by the chosen confidence level, $CL = 1-\alpha$, which is represented by the green area of the background only hypothesis $p_b(Q)$. The blue area represents the Type-II error, $1-\beta$, and meets the condition $1-\beta = \alpha$ satisfied at the exclusion threshold, which is s for this figure. Signals stronger than the threshold $s' > s$ represent models within the exclusion region, dashed-blue lines.

Our background only hypothesis is the likelihood, Eq. (4), with $A_s = 0$ giving a detection threshold at a confidence level $CL = 1-\alpha$

$$1-\alpha = \int_0^{A^{\rm dt}} \mathcal{L}\left(A\middle|A_s = 0\right) dA = \int_0^{A^{\rm dt}} 2Ae^{-A^2} dA \qquad \Longrightarrow \qquad A^{\rm dt} = \sqrt{\ln\left(1/\alpha\right)}. \tag{26}$$

The goal is to ensure that false exclusion of the true signal value, $1-\beta$, does not occur more often than the Type-I error, $1-\beta \leq \alpha$, for any signal strength above a certain threshold. The threshold signal strength, $A_s^{\rm th}$, at $1-\beta = \alpha$ determines the constraint at a confidence CL (or $\lesssim \alpha$ for discontinuous distributions). So, for the signal plus background case

$$1-\beta = \int_0^{A^{\rm dt}} \mathcal{L}\left(A\middle|A_s = A_s^{\rm th}\right) dA, \tag{27}$$

which yields a limit at the 95% CL ($\alpha = 0.05$) in terms of γ of

$$\gamma_{95\%}^{\rm det} = 5.6 \frac{\sigma}{\sqrt{N}\xi\Phi_{\rm DM}}. \tag{28}$$

This limit agrees with the Bayesian approach within 5% using the Berger-Bernardo prior and within 1% using the uniform prior.

To treat the stochastic case we repeat the above calculation using the marginal likelihood, Eq. (11). There is some debate behind treating the likelihood as a PDF in this fashion [12], but it is occasionally done in frequentist approaches [16] and produces reasonable results in our case.

Repeating the previous derivation replacing $\mathcal{L}$ with $\mathcal{L}_m$ yields the constraint

$$\gamma_{95\%}^{\rm stoch} = 15.1 \frac{\sigma}{\sqrt{N}\xi\Phi_{\rm DM}}. \tag{29}$$

This result is within 3% of the Bayesian approach with the Berger-Bernado prior, but is ~ 4 times smaller than that using a uniform prior. This constraint agrees with a straightforward Monte Carlo (MC) approach that also yielded reasonable fits to the distributions used in this section.

C. Gradient coupling and DC sensitivity

For the gradient coupling, the particular sensitivity axis, time of the experiment, and assumptions made mean that we are unable to calculate a uniform correction factor to apply across the currently published constraints. Many experimental constraints have neglected the power present in the galactic-rest frame (or perpendicular to the lab frame motion), typically setting it to zero. Here we show that under some reasonable assumptions the correction factor is roughly the same as that to the scalar coupling. However, for experiments that neglected the rest-frame contribution, we recommend a reanalysis of their data.

The signal for a gradient-coupled experiment can be modeled as

$$s(t) = \gamma\vec{\beta}\cdot\nabla\phi(\boldsymbol{r},t), \tag{30}$$

with γ the appropriate coupling constant and $\vec{\beta}$ the unit sensitivity vector. Similar to Eq. (1), for $T \ll \tau_c$, we can describe a component of the gradient as

$$(s(t))_j \approx \gamma\Psi_j\cos(2\pi f_\phi t + \theta), \tag{31}$$

where Ψ_j is the gradient field amplitude (analogous to Φ_0) and we have assumed the sensitivity axis and galactic wave vector are parallel.

Using the results of Ref. [17] we can obtain a distribution for the gradient field amplitude and use it to treat the problem in a similar fashion as in the main text or, as we show here, analogously to Sec. Supplementary Note 1 B. For more details and definitions behind the following equations please see Ref. [17]. The distribution for one component of the gradient (see Eq. 4.12 of Ref. [17]) is

$$\mathrm{d}|\Psi_j|P(|\Psi_j|) = \mathrm{d}|\Psi_j|\frac{|\Psi_j|}{\tilde{\Gamma}_j^2}\exp\left(-\frac{|\Psi_j|^2}{2\tilde{\Gamma}_j^2}\right). \tag{32}$$

which is similarly (to the scalar amplitude) Rayleigh distributed. The shape parameter of the distribution for a particular coordinate, $\tilde{\Gamma}_j$, is defined as

$$\tilde{\Gamma}_j^2 \equiv \frac{1}{2}\sum_{\vec{k}} A_{\vec{k}}^2 k_j^2, \tag{33}$$

with the Fourier amplitudes, $A_{\vec{k}}$, defined in the lab frame as

$$A_{\vec{k}} = \sqrt{\langle\rho\rangle/m}\left(\sqrt{8\pi}/Lk_0\right)^{3/2}\exp\left[-\frac{k_x^2+k_y^2+(k_z-k_{lab})^2}{k_0^2}\right], \tag{34}$$

where k_0 is set to agree with the SHM virial velocity and the lab velocity is chosen to be along the z direction.

We see that $\tilde{\Gamma}$ is the quantity of interest that determines the relative anisotropy for a detector along the different directions. For either axis perpendicular to the lab's motion, $\tilde{\Gamma}_x = \tilde{\Gamma}_y$, we get

$$\tilde{\Gamma}_x^2 = \left(\frac{L}{2\pi}\right)^3\int d^3k A_{\vec{k}}^2 k_x^2 = \frac{1}{2}\left(\frac{k_0^2}{4}\right), \tag{35}$$

and for the direction parallel to the motion

$$\tilde{\Gamma}_z^2 = \left(\frac{L}{2\pi}\right)^3\int d^3k A_{\vec{k}}^2 k_z^2 = \frac{1}{2}\left(\frac{k_0^2}{4}+k_{lab}^2\right). \tag{36}$$

We can now restate the problem for the gradient field amplitude, Ψ, in the same way as Eq. (2) above. Along the z-direction, Ψ_0 (we drop the subscript to simplify the equations) has the following deterministic and stochastic distributions (see Eq. (2) above for the scalar case)

$$p(\Psi_0|\Psi_{\mathrm{DM}}) = \begin{cases} \delta(\Psi_0-\Psi_{\mathrm{DM}}), & \text{deterministic} \\ \frac{2\Psi_0}{5\Psi_{\mathrm{DM}}^2/4}\exp\left(-\frac{\Psi_0^2}{5\Psi_{\mathrm{DM}}^2/4}\right), & \text{stochastic.} \end{cases} \tag{37}$$

We assume the deterministic gradient field amplitude is $\Psi_{\rm DM} = \sqrt{2\rho_{\rm DM}}v_{\rm lab}$ and that $v_{\rm lab} \approx v_{\rm vir}$. The factor of 5/4 is introduced since the deterministic assumption neglects the rest-frame contribution (the power proportional to k_0^2). We compare the scalar and gradient field amplitude distributions in Fig. 2.

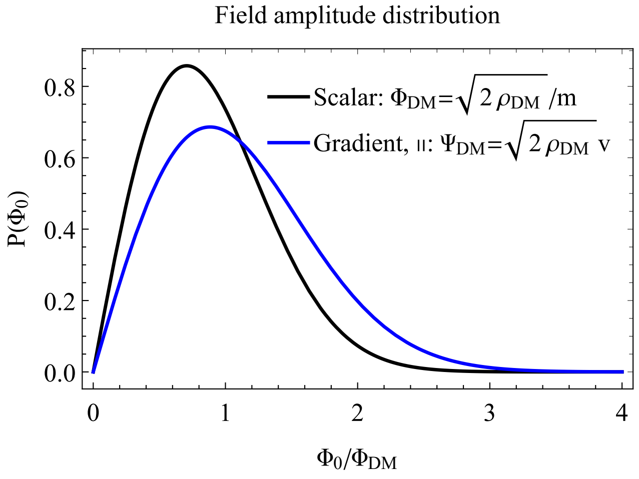

Supplementary Figure 2: Comparison of the gradient and scalar field amplitude distributions.

We can now see it is straightforward to calculate the correction factor using $\mathcal{L}_m\left(A\middle|A_s^{\rm DM}\right)$ and the approach from the previous section with $A_s^{\rm DM}$ defined in the context of the gradient coupling and rescaled by 5/4. This yields a correction factor for the gradient coupling under these simplifying assumptions to be

$$\gamma_{95\%}^{\rm stoch} = 2.2\,\gamma_{95\%}^{\rm det}\,. \tag{38}$$

This correction factor largely depends on the initial assumptions made in the experimental analysis and the detector orientation. Additionally, depending on the frequency regime and duration of the experiment, modulation of the gradient field power will occur at the earth's daily rotation and orbital frequency (one can include this in the k_{lab} term above). We have found that the relative scaling between k_0^2, k_{lab}^2 and the overall factor can vary depending on the chosen field description, for example comparing the extended results of Refs. [1, 17, 18].

1. *Constraints below frequency resolution* ($1/T$)

In this section we briefly discuss marginalization over the unknown phase θ of the VULF and, in particular, the effect of this marginalization on searches for VULFs with frequencies below $1/T$. This problem was the subject of a recent debate in the literature related to the CASPEr-ZULF-Comagnetometer limits originally presented in Ref. [19]. The comment [20] on the published article [19] correctly pointed out that the limits for frequencies below the $1/T$ frequency resolution should not improve compared to limits for frequencies above the $1/T$ frequency resolution. This is true and the mistake in Ref. [19] came from not marginalizing over the uniform distribution of phase, θ in Eq. (31) above, and erroneously assuming the average signal amplitude in this limit $\propto \frac{1}{2\pi}\int|\cos(\theta)|d\theta = 2/\pi$, a deterministic approach to θ similar in principle to the erroneous deterministic approaches to VULF amplitudes and velocities [21]. However, after accounting for this unknown phase as discussed both in the present work and in the reply [21], the authors of the comment [20] still disagreed with our result that the constraint levels off for arbitrarily low frequencies.

The resolution of this debate is rooted in how constraints on couplings of VULF dark matter to standard model particles and fields should be interpreted. Even when assuming that VULF dark matter is well described by the SHM, at a particular time and place, the experimentally observable signal is affected by a number of unknown VULF parameters beyond the coupling strength such as the phase, velocity, and amplitude. Even if the duration T of an experiment is too short to broadly sample the distribution of the unknown VULF parameters, the experiment still excludes the existence of VULF dark matter over some finite volume of a multi-dimensional space of the unknown parameters. To condense the results of a VULF dark matter search into a two-dimensional plot of coupling strength vs. frequency, it is necessary to marginalize over the other unknown VULF parameters. While the authors of the comment [20] object to this marginalization, arguing that for particular phases θ the constraints on couplings could be much weaker, we argue that it is reasonable to plot constraints on couplings ruled out over 95% of the multi-dimensional unknown VULF parameter space. Furthermore, as we note here, the authors of the comment [20] have

treated the amplitude and velocity of the VULF dark matter as deterministic in their own experiment [22]. For particular (but unlikely) VULF velocities and amplitudes, the experiment [22] is insensitive to VULF couplings. In order to constrain VULF dark matter couplings in the regime where T is shorter than either the coherence time or period of the VULF, it is necessary to carry out some form of marginalization over the unknown VULF parameters.

The derived constraints on VULF dark matter presented in Ref. [19] take advantage of the daily modulation provided by earth's rotation. Any gradient-coupled DM signal with frequency f_ϕ below the $1/T$ resolution will be upconverted to the 1/day frequency (f_E) given by Earth's rotation. Thus, instead of trying to resolve frequencies below the $1/T$ resolution, the authors constrain all frequencies below that threshold by looking only at the f_E bin that is within the detection bandwidth. The constraint levels off (becomes constant) for all frequencies below $\sim 1/(25T)$ when $2\pi f_\phi T \approx 0$ and the signal amplitude is dominated by the $|\cos\theta|$ distribution, see Eq. (31). The height of this constant level is determined by the chosen confidence level, as there are a small range of phases that give vanishing signals for arbitrarily large coupling.

This approach can be used for all gradient-coupled ("wind"-type) experiments that can resolve the f_E frequency. We additionally note that scalar searches (for which this upconversion does not occur) which are sensitive to offsets (the zero-frequency component) can also draw similar constraints below the $1/T$ frequency threshold. There is a caveat that any signal present at the f_E frequency would not allow determination of the actual f_ϕ, only that it is smaller than $1/T$. Additionally, differentiating systematic errors from potential VULF dark matter signals is difficult for upconverted frequencies; one approach would be to confirm the expected yearly modulation in the event of a detection.

D. Blueice Monte Carlo

Here we employ a method widely used in astroparticle physics [23] to compare to the Bayesian and frequentist approaches described in the previous sections. In the case encountered here we find that Wilks theorem, the use of a chi-squared distribution for the log likelihood ratio test statistic, does not hold. The reason for this is in part due to the fact that we are not using the profile likelihood but marginalize over the amplitude fluctuation by drawing from the Rayleigh distribution in each MC realization. In a situation like this, one has to resort to doing a so-called Neyman construction, see e.g. discussion in Ref. [16].

The decisive feature of a Neyman construction is that it provides the statistically desired properties (in this case the coverage of the confidence intervals). It uses the distribution of the test statistic as estimated by many MC simulations instead of the asymptotic distribution used in Wilks theorem. For a given signal strength γ, the test-statistic distribution is obtained by running many MC simulations at that signal strength. The critical value is the value of the test statistic at the 90th (or 95th) percentile of the distribution for a test at the 90% (or 95%) confidence level. This procedure is repeated for different signal strengths such that a critical value curve as a function of the signal strength is obtained. The upper limit on the signal strength for a given measurement (or MC simulation) is then given by the value at which the likelihood crosses the critical value curve.

The correction factor is finally not determined from a single experiment but rather from an ensemble of experiments over which the so called sensitivity (median limit in case of no signal) is calculated. The correction factor is then defined analogous to the case of the Bayesian analysis as:

$$correction = \frac{\text{median}(\boldsymbol{u}_{stochastic})}{\text{median}(\boldsymbol{u}_{deterministic})}, \tag{39}$$

where $\boldsymbol{u}$ is a large number of upper limits at a certain confidence level. Initial results from the simulations at the 95% confidence level are similar to those discussed above when including only the field amplitude distribution, see Sec. 6 of Ref. [24] for simulation details. Work on implementing the correct distributions and signal model is ongoing.

Supplementary Note 2. MODIFIED CONSTRAINT PLOT

Here we present a more detailed parameter exclusion plot for the scalar dark matter coupling where we apply a correction factor based on the analysis method used by the respective experiment. We do not do the same for the gradient coupling per our discussion in Sec. Supplementary Note 1 C.

The scalar coupling has only the field amplitude parameter to account for in the correction factor as treated in the main text. We use the result from the marginal likelihood of $\gamma_{95\%}^{\text{stoch}}/\gamma_{95\%}^{\text{det}} = 2.7$ from Sec. Supplementary Note 1 B for the experiments using a frequentist based analysis and 3.0 from Eq. (19) for the experiment using a Bayesian framework [25].

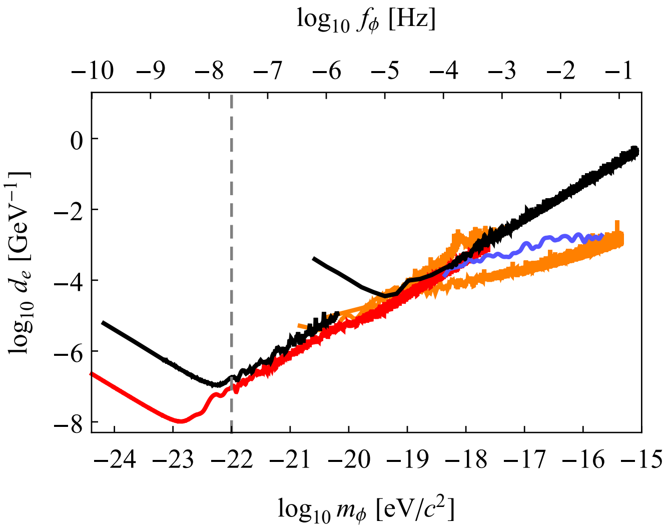

Supplementary Figure 3: An updated VULF exclusion plot of laboratory constraints for dilaton coupling strength d_e. Details on the data for the dual rubidium and cesium cold atom fountain FO2 at LNE-SYRTE, black lines, can be found in Ref. [25], for the dysprosium spectroscopy, red line, in Ref. [26], clock comparisons, orange lines, in Ref. [27], and from the global network of optical atomic clocks, blue line, in Ref [28]. The gray dashed line, $m_\phi \lesssim 10^{-22}$ eV, indicates where the assumption that the field with mass m_ϕ makes up all the dark matter should be relaxed [29].

Supplementary Note 3. LIKELIHOOD

Here we demonstrate the change of notation from Ref. [1] to that used in this paper, and discuss the change of parameters between complex Fourier components, amplitude, and power. Starting with the signal, Eq. (1), and with $\tilde{d}_p$ the DFT component of the original data, the likelihood at f_p is given by

$$\mathcal{L}\left(\tilde{d}_p|\Phi_0,\gamma,\theta\right) = \frac{1}{\pi N\sigma^2}\exp\left\{-\frac{\left|\tilde{d}_p-\tilde{s}_p\right|^2}{N\sigma^2}\right\}, \tag{40}$$

where $\tilde{s}_p = N\gamma\xi\Phi_0 e^{i\theta}/2$ is the DFT component of the signal (1). See Appendix of Ref. [1] for a review. For the phase, one can marginalize over θ as done in Ref. [30], however the resulting likelihood can only be used for Bayesian approaches, see discussion in Ch. 2 and Appendix A of Ref. [31]. To remain consistent with frequentist based approaches we can instead do a change of variables to excess amplitude $A = \left|\tilde{d}_p\right|/\sqrt{N\sigma^2}$. First we rewrite Eq. (40) in polar coordinates using $\tilde{d}_p = |\tilde{d}_p|\exp^{-i\phi}$ and $d\tilde{d}_p = |\tilde{d}_p|d|\tilde{d}_p|d\phi$ as

$$\mathcal{L}\left(|\tilde{d}_p|,\phi\middle|\Phi_0,\gamma,\theta\right) = \frac{|\tilde{d}_p|}{\pi N\sigma^2}\exp\left\{-\frac{|\tilde{d}_p|^2+|\tilde{s}_p|^2}{N\sigma^2}\right\}\exp\left\{\frac{2|\tilde{d}_p||\tilde{s}_p|\cos(\phi-\theta)}{N\sigma^2}\right\}, \tag{41}$$

and then do a vector to scalar, $(|\tilde{d}_p|,\phi)\to A$, change of variables

$$\mathcal{L}\left(A|A_s\right) = \int d|\tilde{d}_p|\int d\phi\ \delta\left(A-\frac{|\tilde{d}_p|}{\sqrt{N\sigma^2}}\right)\mathcal{L}\left(|\tilde{d}_p|,\phi|\Phi_0,\gamma,\theta\right) \tag{42}$$

$$= \int d|\tilde{d}_p|\int d\phi\ \sqrt{N\sigma^2}\ \delta\left(A\sqrt{N\sigma^2}-|\tilde{d}_p|\right)\mathcal{L}\left(|\tilde{d}_p|,\phi|\Phi_0,\gamma,\theta\right) \tag{43}$$

$$= 2Ae^{-A^2-A_s^2}I_0\left(2AA_s\right), \tag{44}$$

with excess signal amplitude $A_s = |\tilde{s}_p|/\sqrt{N\sigma^2}$ and I_0 is the modified Bessel function of the first kind.

It is also common to work with the power spectral density [3] and excess power, $P = |\tilde{d}_p|^2/(N\sigma^2)$, where another change of variables yields

$$\mathcal{L}\left(P \middle| P_s\right) = e^{-(P+P_s)} I_0\left(2\sqrt{PP_s}\right) , \tag{45}$$

with P_s defined accordingly. See Ref. [3] for the original and alternate derivation of Eq. (45).

Supplementary Note 4. COHERENCE TIME

Another topic is the presence of various definitions of coherence time used throughout the literature. The most common definition used is $\tau_c \equiv \left(f_c v_{\rm vir}^2/c^2\right)^{-1}$ and is the one used in this paper. It is a lower bound derived by considering our motion through the spatial gradients of the field [32, 33] given by λ, the De Broglie wavelength,

$$\tau_c \sim \lambda/v_{\rm vir} = \frac{h}{m_\phi v_{\rm vir}^2} = \left(f_c v_{\rm vir}^2/c^2\right)^{-1} \approx 10^6/f_c = 1s\left(\frac{\rm MHz}{m_\phi}\right), \tag{46}$$

where we have used the Compton frequency $f_c = m_\phi c^2/h$ and $v_{\rm vir} \approx 10^{-3}c$. However, some definitions differ by factors around $\sim 2\pi$ [1]. A factor of 2 can occur from using the kinetic energy $mv^2/2$ instead of mv^2 as in Ref. [32, 33] or in using 10^6 as the quality factor and corresponding definition of coherence time.

These differences in coherence time do not have a significant effect on the results in this paper nor previous published constraints. The strongest effect would be the shift of the upper bound on m_a of the CASPEr-ZULF-Sidebands constraint. Since the experiment is incoherently averaging past this point the constraint significantly weakens and was not reported. In addition, the fuzzy definition of coherence time also depends on the relative velocity, making it a distributed quantity.

Regarding this upper bound, a potentially more precise $T_{\rm max}$ rather than the coherence time can be derived (where the regime discussed in this paper would go from $T \ll \tau_c$ to $T \ll T_{\rm max}$) considering the lineshape derived in Ref. [1]. Using the derived full width at half maximum $\Delta\omega \approx 2.5/\tau_c$ and setting it equal to the DFT frequency step $\delta\omega = 2\pi/T$ gives $T_{\rm max} \approx 2\pi\tau_c/2.5$, or with the definition of coherence time used in this paper: $T_{\rm max} \approx \tau_c/2.5$. Thus it is safe to assume the data from the experiments shown are all in the $T \ll T_{\rm max}$ regime, regardless of the definition of coherence time.

Supplementary Note 5. RAYLEIGH DISTRIBUTION

As shown in Eq. (1), Φ_0 is the observed field amplitude, and in the case of total interrogation time being much less than the field's coherence time, $T \ll \tau_c$, the only field amplitude observed during a measurement. One can relate the local dark matter density $\rho_{\rm DM}$ to the field's energy density $\Phi_0^2 f_\phi^2/2 = \rho_{\rm DM}$ as done for example in Ref. [32]. The problem with this is that Φ_0 is actually a Rayleigh distributed value (approximately, see the following subsection), thus the correct expression is

$$\langle\Phi_0^2\rangle \frac{c^2 m_\phi^2}{2\hbar^2} = \rho_{\rm DM} \,. \tag{47}$$

We can define $\Phi_{\rm DM}$ as the value satisfying Eq. (47)

$$\Phi_{\rm DM} = \sqrt{\langle\Phi_0^2\rangle} = \frac{\sqrt{2\rho_{\rm DM}}}{m_\phi}, \tag{48}$$

analogous to previous assumptions of $\Phi_0 = \Phi_{\rm DM}$ in the deterministic approach.

Using Eq. (47) we can solve for the required σ of the Rayleigh distribution $p(\Phi_0)$.

$$\langle\Phi_0^2\rangle = \int \Phi_0^2 p(\Phi_0) d\Phi_0 = \int_0^\infty \frac{\Phi_0^3}{\sigma^2} \exp\left(-\frac{\Phi_0^2}{2\sigma^2}\right) d\Phi_0 = \Phi_{\rm DM}^2$$
$$\implies \sigma = \Phi_{\rm DM}/\sqrt{2} \text{ thus,}$$
$$p(\Phi_0) = 2\frac{\Phi_0}{\Phi_{\rm DM}^2} \exp\left(-\frac{\Phi_0^2}{\Phi_{\rm DM}^2}\right) . \tag{49}$$

Note that the average value of this distribution $\langle\Phi_0\rangle = \Phi_{\rm DM}\sqrt{\pi}/2$ is not simply $\Phi_{\rm DM}$. Approximately 63% of given field realizations will have a field amplitude smaller than $\Phi_{\rm DM}$. The distribution compared to $\Phi_{\rm DM}$ is shown in Fig. 4.

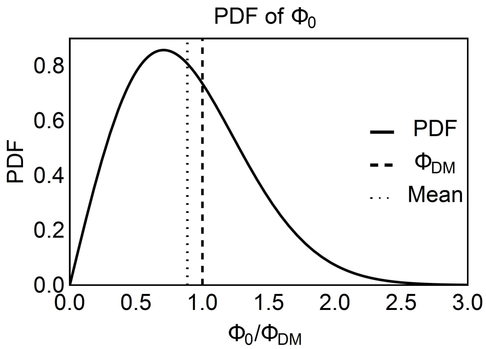

Supplementary Figure 4: Rayleigh distribution given by Eq. (49).

A. Simulated energy distribution

The energy density distribution, Eq. (3), and amplitude distribution, Eq. (49), are only exact under the random phase model. The distribution depends on a number of parameters such as galaxy mass and position within the galaxy [17]. As shown in Ref. [17] simulations of these distributions indicate a slightly heavier tail with a best fit distribution $\rho_{\rm DM} p(\rho) = 0.1 e^{-0.42\rho/\rho_{\rm DM}} + 0.9 e^{-1.06\rho^2/\rho_{\rm DM}^2}$. We compare the two distributions below, where a slightly heavier tail is evident in the log-log plot. Marginalizing over this distribution instead of the random phase model, following the steps in Sec. Supplementary Note 1 B, only changes the derived constraint by $\approx 3\%$.

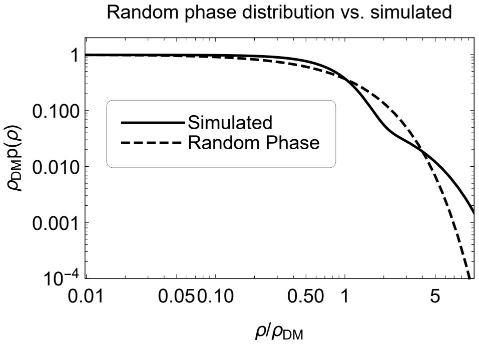

Supplementary Figure 5: Comparison of energy density distribution under the random phase model and from fuzzy dark matter simulations [17].

Further investigation of how gravitational interactions impact these distributions for our particular conditions and if their characteristic shape can be utilized for inference is ongoing.

[1] A. Derevianko, Detecting dark-matter waves with a network of precision-measurement tools, Physical Review A **97**, 042506 (2018), arXiv:1605.09717.

[2] S. Knirck, A. J. Millar, C. A. O'Hare, J. Redondo, and F. D. Steffen, Directional axion detection, Journal of Cosmology and Astroparticle Physics **2018** (11), 051, arXiv:1806.05927.

[3] E. J. Groth, Probability distributions related to power spectra., The Astrophysical Journal Supplement Series **29**, 285 (1975).

[4] C. Dailey, C. Bradley, D. F. Jackson Kimball, I. A. Sulai, S. Pustelny, A. Wickenbrock, and A. Derevianko, Quantum sensor networks as exotic field telescopes for multi-messenger astronomy, Nature Astronomy **5**, 150 (2021), arXiv:2002.04352.

[5] E. T. Jaynes, Bayesian Spectrum and Chirp Analysis, in *Maximum-Entropy and Bayesian Spectral Analysis and Estimation Problems*, edited by C. R. Smith and G. J. Erickson (D. Reidel Publishing Company, Dordrecht, Holland, 1987) pp. 1–37.

[6] R. D. Cousins, Why isn't every physicist a Bayesian?, American Journal of Physics **63**, 398 (1995).

[7] J. Heinrich, Review of the banff challenge on upper limits, in *PHYSTAT LHC Workshop on Statistical Issues for LHC Physics, PHYSTAT 2007 - Proceedings* (2008).

[8] L. Lyons, Open statistical issues in Particle Physics, The Annals of Applied Statistics **2**, 887 (2008).

[9] I. Narsky, Estimation of upper limits using a Poisson statistic, Nuclear Instruments and Methods in Physics Research Section A: Accelerators, Spectrometers, Detectors and Associated Equipment **450**, 444 (2000).

[10] A. L. Read, MODIFIED FREQUENTIST ANALYSIS OF SEARCH RESULTS (THE CLs METHOD), Cern-Open 10.5170/CERN-2000-005.81 (2000).

[11] A. Gelman, D. Simpson, and M. Betancourt, The Prior Can Often Only Be Understood in the Context of the Likelihood, Entropy **19**, 555 (2017).

[12] J. O. Berger, B. Liseo, and R. L. Wolpert, Integrated likelihood methods for eliminating nuisance parameters, Statistical Science 10.1214/ss/1009211804 (1999).

[13] R. E. Kass and L. Wasserman, The Selection of Prior Distributions by Formal Rules, Journal of the American Statistical Association **91**, 1343 (1996).

[14] J. M. Bernardo, Reference Posterior Distributions for Bayesian Inference, Journal of the Royal Statistical Society: Series B (Methodological) **41**, 113 (1979).

[15] M. G. Kendall and A. Stuart, *The Advanced Theory of Statistics. Vol. 2: Inference and: Relationsship* (Griffin, 1973).

[16] M. Tanabashi, K. Hagiwara, K. Hikasa, K. Nakamura, Y. Sumino, F. Takahashi, J. Tanaka, K. Agashe, G. Aielli, C. Amsler, M. Antonelli, D. M. Asner, H. Baer, S. Banerjee, R. M. Barnett, T. Basaglia, C. W. Bauer, J. J. Beatty, V. I. Belousov, J. Beringer, S. Bethke, A. Bettini, H. Bichsel, O. Biebel, K. M. Black, E. Blucher, O. Buchmuller, V. Burkert, M. A. Bychkov, R. N. Cahn, M. Carena, A. Ceccucci, A. Cerri, D. Chakraborty, M.-C. Chen, R. S. Chivukula, G. Cowan, O. Dahl, G. D'Ambrosio, T. Damour, D. de Florian, A. de Gouvêa, T. DeGrand, P. de Jong, G. Dissertori, B. A. Dobrescu, M. D'Onofrio, M. Doser, M. Drees, H. K. Dreiner, D. A. Dwyer, P. Eerola, S. Eidelman, J. Ellis, J. Erler, V. V. Ezhela, W. Fetscher, B. D. Fields, R. Firestone, B. Foster, A. Freitas, H. Gallagher, L. Garren, H.-J. Gerber, G. Gerbier, T. Gershon, Y. Gershtein, T. Gherghetta, A. A. Godizov, M. Goodman, C. Grab, A. V. Gritsan, C. Grojean, D. E. Groom, M. Grünewald, A. Gurtu, T. Gutsche, H. E. Haber, C. Hanhart, S. Hashimoto, Y. Hayato, K. G. Hayes, A. Hebecker, S. Heinemeyer, B. Heltsley, J. J. Hernández-Rey, J. Hisano, A. Höcker, J. Holder, A. Holtkamp, T. Hyodo, K. D. Irwin, K. F. Johnson, M. Kado, M. Karliner, U. F. Katz, S. R. Klein, E. Klempt, R. V. Kowalewski, F. Krauss, M. Kreps, B. Krusche, Y. V. Kuyanov, Y. Kwon, O. Lahav, J. Laiho, J. Lesgourgues, A. Liddle, Z. Ligeti, C.-J. Lin, C. Lippmann, T. M. Liss, L. Littenberg, K. S. Lugovsky, S. B. Lugovsky, A. Lusiani, Y. Makida, F. Maltoni, T. Mannel, A. V. Manohar, W. J. Marciano, A. D. Martin, A. Masoni, J. Matthews, U.-G. Meißner, D. Milstead, R. E. Mitchell, K. Mönig, P. Molaro, F. Moortgat, M. Moskovic, H. Murayama, M. Narain, P. Nason, S. Navas, M. Neubert, P. Nevski, Y. Nir, K. A. Olive, S. Pagan Griso, J. Parsons, C. Patrignani, J. A. Peacock, M. Pennington, S. T. Petcov, V. A. Petrov, E. Pianori, A. Piepke, A. Pomarol, A. Quadt, J. Rademacker, G. Raffelt, B. N. Ratcliff, P. Richardson, A. Ringwald, S. Roesler, S. Rolli, A. Romaniouk, L. J. Rosenberg, J. L. Rosner, G. Rybka, R. A. Ryutin, C. T. Sachrajda, Y. Sakai, G. P. Salam, S. Sarkar, F. Sauli, O. Schneider, K. Scholberg, A. J. Schwartz, D. Scott, V. Sharma, S. R. Sharpe, T. Shutt, M. Silari, T. Sjöstrand, P. Skands, T. Skwarnicki, J. G. Smith, G. F. Smoot, S. Spanier, H. Spieler, C. Spiering, A. Stahl, S. L. Stone, T. Sumiyoshi, M. J. Syphers, K. Terashi, J. Terning, U. Thoma, R. S. Thorne, L. Tiator, M. Titov, N. P. Tkachenko, N. A. Törnqvist, D. R. Tovey, G. Valencia, R. Van de Water, N. Varelas, G. Venanzoni, L. Verde, M. G. Vincter, P. Vogel, A. Vogt, S. P. Wakely, W. Walkowiak, C. W. Walter, D. Wands, D. R. Ward, M. O. Wascko, G. Weiglein, D. H. Weinberg, E. J. Weinberg, M. White, L. R. Wiencke, S. Willocq, C. G. Wohl, J. Womersley, C. L. Woody, R. L. Workman, W.-M. Yao, G. P. Zeller, O. V. Zenin, R.-Y. Zhu, S.-L. Zhu, F. Zimmermann, P. A. Zyla, J. Anderson, L. Fuller, V. S. Lugovsky, and P. Schaffner, Review of Particle Physics, Physical Review D **98**, 030001 (2018), arXiv:0601168 [astro-ph].

[17] L. Hui, A. Joyce, M. J. Landry, and X. Li, Vortices and waves in light dark matter, Journal of Cosmology and Astroparticle Physics **2021** (01), 011.

[18] J. W. Foster, N. L. Rodd, and B. R. Safdi, Revealing the dark matter halo with axion direct detection, Physical Review D **97**, 123006 (2018).

[19] T. Wu, J. W. Blanchard, G. P. Centers, N. L. Figueroa, A. Garcon, P. W. Graham, D. F. J. Kimball, S. Rajendran, Y. V. Stadnik, A. O. Sushkov, A. Wickenbrock, and D. Budker, Search for Axionlike Dark Matter with a Liquid-State Nuclear Spin Comagnetometer, Physical Review Letters **122**, 191302 (2019), arXiv:1901.10843.

[20] E. G. Adelberger and W. A. Terrano, Comment on "Search for Axionlike Dark Matter with a Liquid-State Nuclear Spin Comagnetometer", Physical Review Letters **123**, 169001 (2019).

[21] T. Wu, J. W. Blanchard, G. P. Centers, N. L. Figueroa, A. Garcon, P. W. Graham, D. F. J. Kimball, S. Rajendran, Y. V. Stadnik, A. O. Sushkov, A. Wickenbrock, and D. Budker, Wu et al. Reply:, Physical Review Letters **123**, 169002 (2019).

[22] W. A. Terrano, E. G. Adelberger, C. A. Hagedorn, and B. R. Heckel, Constraints on Axionlike Dark Matter with Masses Down to 10^{-23} eV/c^2, Physical Review Letters **122**, 231301 (2019).

[23] J. Conrad, Statistical issues in astrophysical searches for particle dark matter (2015).

[24] B. Pelssers, *Enhancing Direct Searches for Dark Matter : Spatial-Temporal Modeling and Explicit Likelihoods (PhD dissertation)*, Ph.D. thesis, Stockholm University (2020).

[25] A. Hees, J. Guéna, M. Abgrall, S. Bize, and P. Wolf, Searching for an Oscillating Massive Scalar Field as a Dark Matter Candidate Using Atomic Hyperfine Frequency Comparisons, Physical Review Letters **117**, 061301 (2016).

[26] K. Van Tilburg, N. Leefer, L. Bougas, and D. Budker, Search for Ultralight Scalar Dark Matter with Atomic Spectroscopy, Physical Review Letters **115**, 011802 (2015).

[27] C. J. Kennedy, E. Oelker, J. M. Robinson, T. Bothwell, D. Kedar, W. R. Milner, G. E. Marti, A. Derevianko, and J. Ye, Precision Metrology Meets Cosmology: Improved Constraints on Ultralight Dark Matter from Atom-Cavity Frequency Comparisons, Physical Review Letters **125**, 201302 (2020).

[28] P. Wcisło, P. Ablewski, K. Beloy, S. Bilicki, M. Bober, R. Brown, R. Fasano, R. Ciuryło, H. Hachisu, T. Ido, J. Lodewyck, A. Ludlow, W. McGrew, P. Morzyński, D. Nicolodi, M. Schioppo, M. Sekido, R. Le Targat, P. Wolf, X. Zhang, B. Zjawin,

and M. Zawada, New bounds on dark matter coupling from a global network of optical atomic clocks, Science Advances **4**, eaau4869 (2018).
[29] D. J. Marsh, Axion cosmology, Physics Reports **643**, 1 (2016), arXiv:0610440 [astro-ph].
[30] E. T. Jaynes, Bayesian Spectrum and Chirp Analysis, in *Maximum-Entropy and Bayesian Spectral Analysis and Estimation Problems* (Springer Netherlands, Dordrecht, 1987) pp. 1–37.
[31] G. L. Bretthorst, *Journal of the American Statistical Association*, Lecture Notes in Statistics, Vol. 48 (Springer New York, New York, NY, 1988) p. 258.
[32] P. W. Graham and S. Rajendran, New observables for direct detection of axion dark matter, Physical Review D - Particles, Fields, Gravitation and Cosmology **88**, 1 (2013), arXiv:arXiv:1306.6088v2.
[33] D. Budker, P. W. Graham, M. Ledbetter, S. Rajendran, and A. O. Sushkov, Proposal for a Cosmic Axion Spin Precession Experiment (CASPEr), Physical Review X **4**, 021030 (2014), arXiv:1306.6089.
